# Exposure to COVID-19-Related Information and its Association With Mental Health Problems in Thailand: Nationwide, Cross-sectional Survey Study

**DOI:** 10.2196/25363

**Published:** 2021-02-12

**Authors:** Pajaree Mongkhon, Chidchanok Ruengorn, Ratanaporn Awiphan, Kednapa Thavorn, Brian Hutton, Nahathai Wongpakaran, Tinakon Wongpakaran, Surapon Nochaiwong

**Affiliations:** 1 Division of Pharmacy Practice, Department of Pharmaceutical Care; Unit of Excellence on Research in Health Outcomes and Patient Safety in Elderly School of Pharmaceutical Sciences University of Phayao Phayao Thailand; 2 Pharmacoepidemiology and Statistics Research Center Faculty of Pharmacy Chiang Mai University Chiang Mai Thailand; 3 Department of Pharmaceutical Care Faculty of Pharmacy Chiang Mai University Chiang Mai Thailand; 4 School of Epidemiology and Public Health Faculty of Medicine University of Ottawa Ottawa, ON Canada; 5 Institute of Clinical and Evaluative Sciences ICES uOttawa Ottawa, ON Canada; 6 Ottawa Hospital Research Institute Ottawa Hospital Ottawa, ON Canada; 7 Department of Psychiatry Faculty of Medicine Chiang Mai University Chiang Mai Thailand

**Keywords:** coronavirus, COVID-19, insomnia, mental health, social media, depression, anxiety, stress, psychosocial problem

## Abstract

**Background:**

The COVID-19 pandemic has had a negative impact on both the physical and mental health of individuals worldwide. Evidence regarding the association between mental health problems and information exposure among Thai citizens during the COVID-19 outbreak is limited.

**Objective:**

This study aimed to explore the relationship between information exposure and mental health problems during the COVID-19 pandemic in Thailand.

**Methods:**

Between April 21 and May 4, 2020, we conducted a cross-sectional, nationwide online survey of the general population in Thailand. We categorized the duration of exposure to COVID-19-related information as follows: <1 h/day (reference group), 1-2 h/day, and ≥3 h/day. Mental health outcomes were assessed using the Patient Health Questionnaire-9, the Generalized Anxiety Disorder-7 scale, the Perceived Stress Scale-10, and the Insomnia Severity Index for symptoms of depression, anxiety, perceived stress, and insomnia, respectively. Multivariable logistic regression models were used to evaluate the relationship between information exposure and the risk of developing the aforementioned symptoms. An ancillary analysis using multivariable multinomial logistic regression models was also conducted to assess the possible dose-response relationship across the severity strata of mental health problems.

**Results:**

Of the 4322 eligible participants, 4004 (92.6%) completed the online survey. Of them, 1481 (37.0%), 1644 (41.1%), and 879 (22.0%) participants were exposed to COVID-19-related information for less than 1 hour per day, 1 to 2 hours per day, or 3 or more hours per day, respectively. The major source of information related to the COVID-19 pandemic was social media (95.3%), followed by traditional media (68.7%) and family members (34.9%). Those exposed to information for 3 or more hours per day had a higher risk of developing symptoms of depression (adjusted odds ratio [OR] 1.35, 95% CI 1.03-1.76; *P*=.03), anxiety (adjusted OR 1.88, 95% CI 1.43-2.46; *P*<.001), and insomnia (adjusted OR 1.52, 95% CI 1.17-1.97; *P*=.001) than people exposed to information for less than 1 hour per day. Meanwhile, people exposed to information for 1 to 2 hours per day were only at risk of developing symptoms of anxiety (adjusted OR 1.35, 95% CI 1.08-1.69; *P*=.008). However, no association was found between information exposure and the risk of perceived stress. In the ancillary analysis, a dose-response relationship was observed between information exposure of 3 or more hours per day and the severity of mental health problems.

**Conclusions:**

These findings suggest that social media is the main source of COVID-19-related information. Moreover, people who are exposed to information for 3 or more hours per day are more likely to develop psychological problems, including depression, anxiety, and insomnia. Longitudinal studies investigating the long-term effects of COVID-19-related information exposure on mental health are warranted.

## Introduction

On December 31, 2019, the world witnessed the occurrence of a new public health emergency, the COVID-19 outbreak, in Wuhan, China [1]. On March 11, 2020, the World Health Organization (WHO) declared COVID-19 a pandemic due to the rapid global spread of the causative virus [2]. As of January 6, 2021, approximately 86 million confirmed cases and over 1.8 million deaths due to COVID-19 were reported worldwide. In addition to the physical effects of COVID-19, the COVID-19 pandemic has negatively affected the mental health of the public globally [3-5].

During the outbreak, people may need some information from the media to better understand the situation and determine strategies to protect their health. Information-seeking behavior may reduce anxiety caused by uncertainty during a disease outbreak or disaster [6]. In contrast, excessive consumption of information provided by the media may create new problems. Large volumes of information may amplify the perception of risk, and consumption of fear-related information may have a negative impact on consumers who cannot discern real news from fake news or cannot obtain a more balanced view of the media coverage of said event [7]. This infodemic has the potential to affect the population’s mental health and well-being. Many previous studies have illustrated that media exposure is associated with adverse psychological outcomes in different contexts, including bioterrorism [8], war [9], natural disasters [10], and mental health of the general population [11].

Since the start of the COVID-19 pandemic, people have been highly dependent on information from the media, especially those who are not directly affected by the disease. People who are quarantined or isolated may also experience psychological problems due to the widespread media coverage related to the COVID-19 outbreak as well as financial difficulties. In addition, patients with confirmed or suspected COVID-19 may experience disease progression and transmit the virus to their families and friends. Numerous studies have investigated the association between COVID-19-related information exposure and mental health [12-17]. A previous study conducted in Wuhan demonstrated that social media exposure was positively associated with anxiety and depression during the COVID-19 outbreak, after controlling for covariates [4]. These results were supported by those of studies conducted in Germany [18], Saudi Arabia [19], and China [20] indicating that the frequency and duration of media or information exposure may predispose individuals to mental distress. These findings highlight the need to address mental health problems as part of public health policy.

In Thailand, the government announced a lockdown on March 26, 2020, in an effort to stop the spread of COVID-19. This measure seemed to have greatly prevented or slowed down the nationwide spread of the disease. However, during this lockdown, people who were quarantined or isolated might have developed psychological distress and other mental health problems due to media information overload and fear of the effects of COVID-19. To our knowledge, no study has reported the relationship between information exposure and mental health in the general population in Thailand during the COVID-19 outbreak. Therefore, we conducted a cross-sectional nationwide online survey to investigate the relationship between information exposure and symptoms of depression, anxiety, stress, and insomnia during the COVID-19 pandemic in Thailand.

## Methods

### Study Design and Study Population

The *Health Outcomes and Mental Health Care Evaluation Survey: Under the Pandemic Situation of COVID-19 (HOME-COVID-19)* was a cross-sectional online survey administered via the SurveyMonkey platform. The HOME-COVID-19 study was specifically developed by our research group and comprised baseline sociodemographic characteristics and a set of measurement tools for evaluating mental health and psychosocial problems; we used the Thai versions of the validated measurement tools or tools developed by our team. The questionnaire was initially revised by a panel of health care professionals, including three epidemiologists, two psychiatrists, one social scientist, and two hospital directors. This was further validated by a pilot survey of 30 health care professionals and 30 individuals from the general population. Further details about the HOME-COVID-19 study have been provided in a previous study [21]. This was a nationwide survey conducted between April 21 and May 4, 2020. This survey was performed by first selecting a sample of individuals from the general population in Thailand. Convenience sampling and a snowball strategy were used for participant recruitment through various social media networks (ie, public websites, Facebook, LINE, Twitter, and Instagram). The characteristics of the target population and the questionnaire used were subsequently presented [21]. We included Thai citizens, permanent residents, and nonresidents with employment or work permits who could read and communicate in the Thai language and were at least 18 years of age during the survey period. We excluded individuals who lacked internet access, were unable to complete the online survey, and spent less than 2 minutes, or more than 60 minutes, completing the survey, which likely made their data invalid. Based on the HOME-COVID-19 survey protocol [21], the participants spent approximately 20 to 30 minutes completing the survey.

Written consent was obtained from the participants in the first section of the online survey before completing the questionnaire. This study was approved by the Research Ethics Committees of the Faculties of Public Health (ET010/2020) and Pharmacy (23/2563), Chiang Mai University, Thailand. The study was reported in accordance with the Strengthening the Reporting of Observational Studies in Epidemiology statement [22] and the Checklist for Reporting Results of Internet E-Surveys guidelines [23].

### Measurements

#### COVID-19-Related Information Exposure

Information on the duration of information exposure was obtained by asking the participants how often they were exposed to news and information about the COVID-19 pandemic. The duration of information exposure was categorized as follows: <1 h/day, 1-2 h/day, or ≥3 h/day. Sources of information were classified as traditional media (eg, newspaper, television, radio, etc), social media (eg, Facebook, Twitter, blogs, etc), colleagues and neighbors, family members, government organizations (eg, Ministry of Health), and others.

#### Mental Health Outcomes

The mental health outcomes of interest included symptoms of depression, anxiety, perceived stress, and insomnia during the COVID-19 pandemic. The aforementioned symptoms were assessed using the Thai versions of the validated measurement tools. The measurement tools used in this study were as follows:

1. The Patient Health Questionnaire-9 (PHQ-9) consists of nine items that reflect the severity of depression symptoms. The score ranges from 0 to 27, and an overall PHQ-9 score of 9 or higher indicates depression (see Multimedia Appendix 1) [24].
2. The Generalized Anxiety Disorder-7 (GAD-7) scale comprises seven items that reflect the severity of anxiety symptoms. The total score ranges from 0 to 21, and a GAD-7 score of 5 or higher indicates anxiety (see Multimedia Appendix 2) [25].
3. The Perceived Stress Scale-10 (PSS-10) is a 5-point Likert scale consisting of 10 items. The scores range from 0 to 40, and a score of 14 or higher indicates perceived stress (see Multimedia Appendix 3) [26].
4. The Insomnia Severity Index (ISI) is an 8-item scale used to assess the nature, severity, and impact of insomnia. The scores range from 0 to 28 points and vary based on the severity of insomnia. In our study, an ISI score of 7 or higher indicated insomnia symptoms (see Multimedia Appendix 4) [27].

### Power

Using the HOME-COVID-19 survey protocol, the target sample size for this study was estimated based on the findings of previous studies conducted during the COVID-19 outbreak, which reported a prevalence rate of mental health and psychosocial problems of 3.3% to 75.5% [21]. To compensate for a design effect of 2.0 and an estimated response rate of 60%, a minimum sample size of 2492 participants was obtained to achieve a power of 80% and a type I error α level of .05. However, there was no restriction on the maximum number of participants in the online survey.

### Statistical Analysis

Descriptive analyses were performed to describe the characteristics of participants among the three information exposure groups. Categorical variables were expressed as the number (%), while continuous variables were expressed as the mean (SD) or median (IQR), as appropriate. Intergroup differences in the duration of COVID-19-related information exposure were tested using the χ^2^ or Fisher exact tests; analysis of covariance and the Kruskal-Wallis test were used to evaluate categorical and continuous variables, respectively.

For the primary analysis, multivariable logistic regression models were used to explain the association between the duration of daily information exposure and the risk of depression (PHQ-9 score 9), anxiety (GAD-7 score 5), perceived stress (PSS-10 score 14), and insomnia (ISI score 8) during the COVID-19 outbreak, after controlling for the covariates of each outcome. The covariates included age, sex, marital status, education level, religion, occupation, region of residence, living status, reimbursement scheme, mental illness history, chronic noncommunicable disease history, income loss, financial problems, confirmed cases in the community, working from home, quarantine status, fear of COVID-19, and resilient coping. The degree of fear against COVID-19 was measured using a 10-point rating scale. The scores were then grouped as follows: no or minimal fear (0-3 points), moderate fear (4-6 points), and severe fear (7-10 points). Resilient coping was defined using the Brief Resilient Coping Scale: low-resilient copers (4-13 points), moderate-resilient copers (14-16 points), and high-resilient copers (17-20 points) [28].

An auxiliary analysis was conducted using multivariable multinomial logistic regression models to explain the association between the duration of information exposure and the severity of mental health problems. Sensitivity analysis was performed using a multivariable linear regression model to confirm the linear relationship between the duration of COVID-19-related information exposure and the risk of mental health problems. Meanwhile, a multivariable ordinal logistic regression model was used to examine the association between the duration of information exposure and severity strata of each mental health problem while controlling for the aforementioned covariates.

The effect estimates were presented as odds ratios (ORs), along with their corresponding 95% CIs, and weighted to match the estimates for the national population and internet users in all models based on data from the National Statistical Office under the Ministry of Information and Communication Technology. All data were analyzed using Stata, version 14.0 (StataCorp LP). A 2-tailed test with *P*<.05 was considered statistically significant.

## Results

### Baseline Characteristics

**Figure 1:**
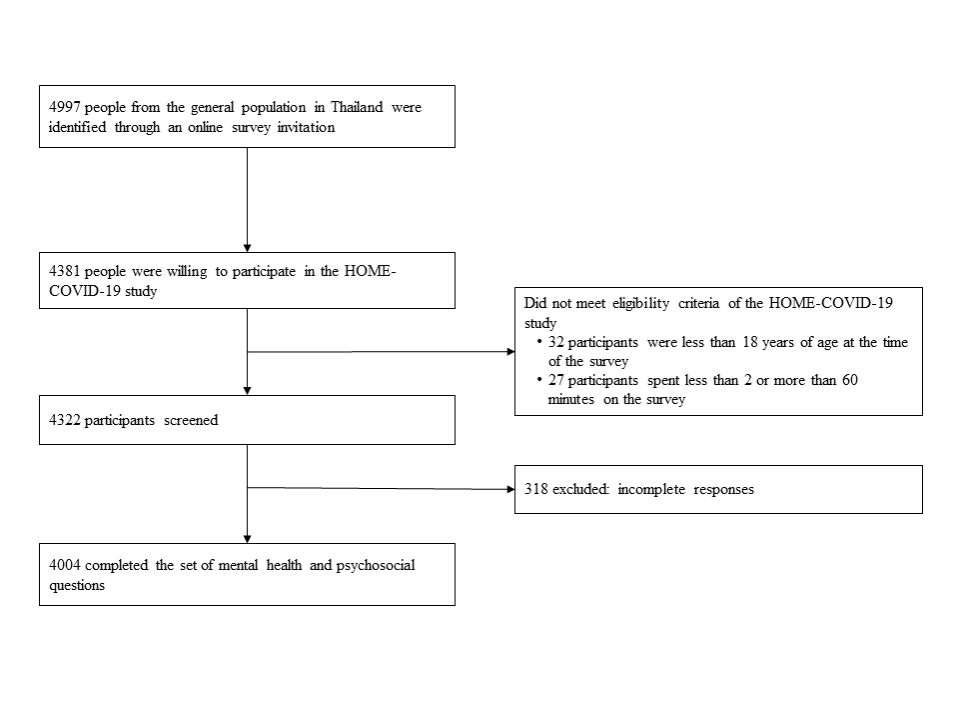
Flow diagram for study participants. HOME-COVID-19: Health Outcomes and Mental Health Care Evaluation Survey: Under the Pandemic Situation of COVID-19.

A total of 4997 participants were invited to complete an online survey, but only 4381 participants responded to the survey. Among 4381 participants, 59 individuals who were under 18 years or age at the time of the survey and/or spent less than 2 minutes, or more than 60 minutes, completing the survey were excluded. Of the 4322 participants who met the eligibility criteria, 4004 completed the online survey (see Figure 1). Of the 4004 participants who completed the survey, 2619 (65.4%) were female, 1231 (30.7%) were male, and 154 (3.8%) selected *other* for the sex question. The mean age was 29.1 (SD 10.8) years; moreover, 3208 (80.1%) participants were single. As shown in Table 1, 127 (3.2%) participants had completed junior high school, while 1893 (47.3%) had completed high vocational education. Among the 4004 participants who completed the survey, 1589 (39.7%) were college students, 526 (13.1%) were government or state enterprise employees, and 500 (12.5%) were private enterprise employees. The participants were mainly from the noncapital city and its environs (2579/4004, 64.4%) in Thailand, whereas 1425 (35.6%) were from the capital city and its environs. Only 383 (9.6%) participants had no fear of COVID-19, whereas 1940 (48.4%) had a severe fear of COVID-19. The majority of the participants were low-resilient copers (1756 /4004, 43.9%), while 678 (16.9%) were highly resilient.

**Table 1:** Participant characteristics and the duration of exposure to COVID-19-related information in Thailand, from April 21 to May 4, 2020.

Participant characteristics			Overall (N=4004), n (%)	Duration of exposure to COVID-19-related information			
				<1 h/day (n=1481)	1-2 h/day (n=1644)	≥3 h/day (n=879)	*P* value
**Sociodemographic data, n (%)**							
	**Age**						
		Total sample (years), mean (SD)	29.1 (10.8)	27.8 (9.8)	28.9 (10.8)	31.5 (12.1)	<.001
		≤30 years	2659 (66.4)	1028 (69.4)	1112 (67.6)	519 (59.0)	<.001
		31-50 years	1088 (27.2)	392 (26.5)	427 (26.0)	269 (30.6)	
		≥51 years	257 (6.4)	61 (4.1)	105 (6.4)	91 (10.4)	
	**Sex**						
		Male	1231 (30.7)	485 (32.8)	500 (30.4)	246 (28.0)	.12
		Female	2619 (65.4)	936 (63.2)	1087 (66.1)	596 (67.8)	
		Other	154 (3.9)	60 (4.0)	57 (3.5)	37 (4.2)	
	**Marital status**						
		Single	3208 (80.1)	1228 (82.9)	1335 (81.2)	645 (73.4)	<.001
		Married or domestic partnership	693 (17.3)	219 (14.8)	277 (16.8)	197 (22.4)	
		Divorced, widowed, or separated	103 (2.6)	34 (2.3)	32 (2.0)	37 (4.2)	
	**Education level**						
		Illiterate, primary school, or junior high school	127 (3.2)	54 (3.6)	46 (2.8)	27 (3.1)	<.001
		Senior high school, diploma, or high vocational school	1893 (47.3)	799 (54.0)	749 (45.6)	345 (39.2)	
		Bachelor’s degree	1559 (38.9)	511 (34.5)	648 (39.4)	400 (45.5)	
		Higher education	425 (10.6)	117 (7.9)	201 (12.2)	107 (12.2)	
	**Religion**						
		Irreligious	375 (9.4)	138 (9.3)	171 (10.4)	66 (7.5)	.23
		Buddhist	3454 (86.3)	1271 (85.8)	1402 (85.3)	781 (88.9)	
		Christian	100 (2.5)	43 (2.9)	42 (2.5)	15 (1.7)	
		Muslim	70 (1.7)	26 (1.8)	28 (1.7)	16 (1.8)	
		Other	5 (0.1)	3 (0.2)	1 (0.1)	1 (0.1)	
	**Occupation**						
		Unemployed or retired	391 (9.8)	130 (8.8)	148 (9.0)	113 (12.9)	<.001
		Farmer or laborer	451 (11.3)	195 (13.2)	156 (9.5)	100 (11.4)	
		Self-employed	415 (10.4)	146 (9.9)	154 (9.4)	115 (13.1)	
		Government or state enterprise employee	526 (13.1)	139 (9.4)	260 (15.8)	127 (14.4)	
		College student	1589 (39.7)	667 (45.0)	659 (40.1)	263 (29.9)	
		Private enterprise employee	500 (12.5)	165 (11.1)	211 (12.8)	124 (14.1)	
		Freelance or other	132 (3.3)	39 (2.6)	56 (3.4)	37 (4.2)	
	**Region of residence**						
		Capital city and its environs	1425 (35.6)	496 (33.5)	623 (37.9)	306 (34.8)	.03
		Noncapital city and its environs	2579 (64.4)	985 (66.5)	1021 (62.1)	573 (65.2)	
	**Living status**						
		Alone	576 (14.4)	195 (13.2)	239 (14.5)	142 (16.2)	.25
		With family	3164 (79.0)	1179 (79.6)	1299 (79.0)	686 (78.0)	
		With others	264 (6.6)	107 (7.2)	106 (6.5)	51 (5.8)	
	**Reimbursement scheme**						
		Government or state enterprises	539 (13.5)	172 (11.6)	254 (15.5)	113 (12.9)	.008
		Universal coverage scheme	1329 (33.2)	524 (35.4)	515 (31.3)	290 (33.0)	
		Social security scheme	1161 (29.0)	423 (28.6)	460 (28.0)	278 (31.6)	
		Self-payment or other	975 (24.3)	362 (24.4)	415 (25.2)	198 (22.5)	
	**History of mental illness**						
		Yes	359 (9.0)	125 (8.4)	142 (8.6)	92 (10.5)	.21
		No	3645 (91.0)	1356 (91.6)	1502 (91.4)	787 (89.5)	
	**History of chronic noncommunicable disease^a^**						
		Yes	599 (15.0)	182 (12.3)	218 (13.3)	199 (22.6)	<.001
		No	3405 (85.0)	1299 (87.7)	1426 (86.7)	680 (77.4)	
**Economic burden and issues with regard to the COVID-19 outbreak, n (%)**							
	**Income loss during the COVID-19 outbreak**						
		Yes	1664 (41.6)	524 (35.4)	660 (40.2)	480 (54.6)	<.001
		No	2340 (58.4)	957 (64.6)	984 (59.8)	399 (45.4)	
	**Financial problems during the COVID-19 outbreak**						
		Yes	2012 (50.2)	668 (45.1)	800 (48.7)	544 (61.9)	<.001
		No	1992 (49.8)	813 (54.9)	844 (51.3)	335 (38.1)	
	**Confirmed cases in the community**						
		No	2562 (64.0)	888 (60.0)	1067 (64.9)	607 (69.1)	<.001
		Yes	641 (16.0)	246 (16.6)	260 (15.8)	135 (15.4)	
		Not known	801 (20.0)	347 (23.4)	317 (19.3)	137 (15.6)	
	**Working from home**						
		Yes	3139 (78.4)	1142 (77.1)	1298 (79.0)	699 (79.5)	.30
		No	865 (21.6)	339 (22.9)	346 (21.0)	180 (20.5)	
	**Quarantine status**						
		Never	1781 (44.5)	610 (41.2)	751 (45.7)	420 (47.8)	.004
		Past	1575 (39.3)	631 (42.6)	636 (38.7)	308 (35.0)	
		Current	648 (16.2)	240 (16.2)	257 (15.6)	151 (17.2)	
	**Fear of COVID-19**						
		None or minimal	383 (9.6)	163 (11.0)	140 (8.5)	80 (9.1)	.04
		Moderate	1681 (42.0)	593 (40.0)	729 (44.3)	359 (40.8)	
		Severe	1940 (48.4)	725 (49.0)	775 (47.1)	440 (50.1)	
	**Resilient coping**						
		Low-resilient coper	1756 (43.9)	683 (46.1)	701 (42.6)	372 (42.3)	.12
		Medium-resilient coper	1570 (39.2)	559 (37.7)	670 (40.8)	341 (38.8)	
		High-resilient coper	678 (16.9)	239 (16.1)	273 (16.6)	166 (18.9)	

^a^Includes diabetes mellitus, hypertension, dyslipidemia, stroke and heart disease, chronic kidney disease, chronic lung disease, and cancer.

### Information Exposure

**Figure 2:**
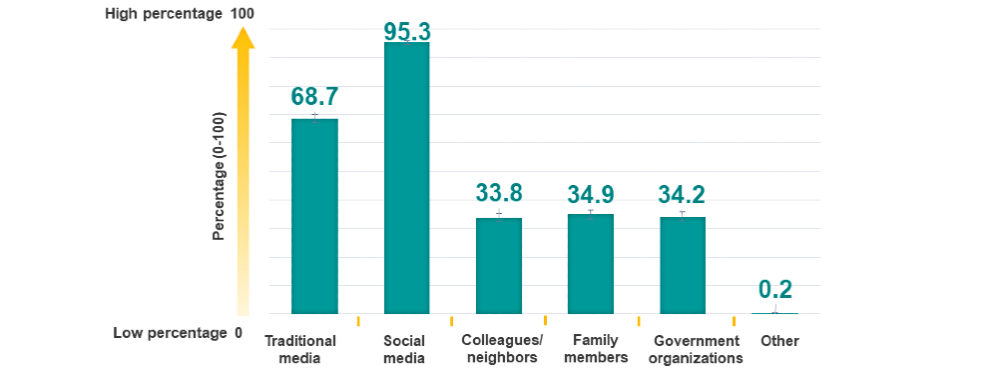
Sources of COVID-19-related information in Thailand. One person can be exposed to more than one source.

Of the 4004 participants, 1644 (41.1%) were exposed to information 1 to 2 hours per day, followed by less than 1 hour per day (1481/4004, 37.0%) and 3 or more hours per day (879/4004, 22.0%). The sources of COVID-19-related information were social media (95.3%, 95% CI 94.6-95.9), followed by traditional media (68.7%, 95% CI 67.2-70.1), and family members (34.9%, 95% CI 33.4-36.4) (see Figure 2).

### Mental Health and Information Exposure

Table 2 presents the results of the multivariable logistic regression analysis that was performed to explain the association between the duration of information exposure and the risk of mental health problems during the COVID-19 pandemic, after controlling for covariates. In the primary analysis, participants who were exposed to COVID-19-related information for 3 or more hours per day had higher odds of developing symptoms of depression than those exposed to COVID-19-related information for less than 1 hour per day (adjusted OR 1.35, 95% CI 1.03-1.76; *P*=.03). Similarly, participants who were exposed to COVID-19-related information for 3 or more hours per day were more likely to develop anxiety and insomnia than those exposed to COVID-19-related information for less than 1 hour per day (adjusted OR 1.88, 95% CI 1.43-2.46; *P*<.001; and adjusted OR 1.52, 95% CI 1.17-1.97; *P*=.001, respectively). Meanwhile, participants who were exposed to COVID-19-related information for 1 to 2 hours per day only had a risk of developing anxiety symptoms (adjusted OR 1.35, 95% CI 1.08-1.69; *P*=.008). No association was observed between the duration of information exposure and the risk of perceived stress in the general population during the study period in Thailand.

**Table 2:** Mental health outcomes according to the duration of exposure to COVID-19-related information: multivariable logistic regression model.

Symptoms developed and duration of exposure to COVID-19-related information		Number of cases, n (%)	Adjusted OR^a^ (95% CI)^b^	*P* value
**Depression: Patient Health Questionnaire-9**				
	<1 h/day (n=1481)	567 (38.3)	Reference (1.00)	N/A^c^
	1-2 h/day (n=1644)	696 (42.3)	1.08 (0.86-1.35)	.52
	≥3 h/day (n=879)	391 (44.5)	1.35 (1.03-1.76)	.03
**Anxiety: Generalized Anxiety Disorder-7**				
	<1 h/day (n=1481)	578 (39.0)	Reference (1.00)	N/A
	1-2 h/day (n=1644)	701 (42.6)	1.35 (1.08-1.69)	.008
	≥3 h/day (n=879)	446 (50.7)	1.88 (1.43-2.46)	<.001
**Stress: Perceived Stress Scale-10**				
	<1 h/day (n=1481)	1076 (72.7)	Reference (1.00)	N/A
	1-2 h/day (n=1644)	1200 (73.0)	0.96 (0.74-1.23)	.72
	≥3 h/day (n=879)	640 (72.8)	1.01 (0.75-1.36)	.93
**Insomnia: Insomnia Severity Index**				
	<1 h/day (n=1481)	760 (51.3)	Reference (1.00)	
	1-2 h/day (n=1644)	877 (53.3)	1.04 (0.84-1.29)	.71
	≥3 h/day (n=879)	520 (59.2)	1.52 (1.17-1.97)	.001

^a^OR: odds ratio.

^b^ORs and corresponding 95% CIs are presented as weighted according to the national population and internet users in Thailand. ORs were adjusted for age, sex, marital status, education level, religion, occupation, region of residence, living status, reimbursement scheme, history of mental illness, history of chronic noncommunicable disease, income loss, financial problems, confirmed cases in the community, working from home, quarantine status, fear of COVID-19, and resilient coping.

^c^N/A: not applicable.

In an ancillary analysis, using a multivariable multinomial logistic regression model (<1 h/day of information exposure [reference group]), a dose-response relationship was noted between exposure to COVID-19-related information for 3 or more hours per day and the severity of mental health problems, particularly anxiety symptoms (see Table 3). For instance, individuals exposed to COVID-19-related information for 3 or more hours per day demonstrated substantial effect modification in terms of the severity strata of anxiety symptoms: the adjusted ORs were 1.35 (95% CI 0.99-1.83; *P*=.06), 2.87 (95% CI 1.91-4.33; *P*<.001), and 4.45 (95% CI 2.45-8.08; *P*<.001) for mild, moderate, and severe anxiety symptoms, respectively.

**Table 3:** Multivariable multinomial logistic regression model results of the duration of exposure to COVID-19-related information and severity of mental health problems.

Severity of mental health problems		Number of cases (N=4004), n (%)	Duration of exposure to COVID-19-related information								
			1-2 h/day					≥3 h/day			
			Estimated adjusted OR^a^ (95% CI)^b^		*P* value		Estimated adjusted OR (95% CI)^b^			*P* value	
**Depression: Patient Health Questionnaire-9**											
	None or minimal (<9 points)	2350 (58.7)	Reference (1.00)	N/A^c^		Reference (1.00)			N/A		
	Mild (9-14 points)	1024 (25.6)	1.05 (0.82-1.34)	.70		1.18 (0.86-1.60)			.31		
	Moderate (15-19 points)	389 (9.7)	1.10 (0.76-1.60)	.60		1.49 (0.97-2.28)			.07		
	Severe (≥20 points)	241 (6.0)	1.13 (0.70-1.84)	.61		2.23 (1.27-3.92)			.005		
**Anxiety: Generalized Anxiety Disorder-7**											
	None or minimal (<5 points)	2279 (56.9)	Reference (1.00)	N/A		Reference (1.00)			N/A		
	Mild (5-9 points)	1117 (27.9)	1.31 (1.03-1.66)	.03		1.35 (0.99-1.83)			.06		
	Moderate (10-14 points)	411 (10.3)	1.33 (0.91-1.93)	.14		2.87 (1.91-4.33)			<.001		
	Severe (≥15 points)	197 (4.9)	1.79 (1.06-3.02)	.03		4.45 (2.45-8.08)			<.001		
**Stress: Perceived Stress Scale-10**											
	None or low (<14 points)	1088 (27.2)	Reference (1.00)	N/A		Reference (1.00)			N/A		
	Moderate (14-26 points)	2550 (63.7)	0.93 (0.72-1.20)	.59		0.96 (0.71-1.30)			.80		
	High (≥27 points)	366 (9.1)	1.22 (0.80-1.88)	.36		1.76 (1.09-2.85)			.02		
**Insomnia: Insomnia Severity Index**											
	None or minimal (<8 points)	1847 (46.1)	Reference (1.00)	N/A		Reference (1.00)			N/A		
	Mild (8-14 points)	1576 (39.4)	0.99 (0.79-1.24)	.94		1.41 (1.07-1.86)			.02		
	Moderate (15-21 points)	486 (12.1)	1.29 (0.91-1.83)	.15		2.03 (1.34-3.07)			.001		
	Severe (≥22 points)	95 (2.4)	0.89 (0.45-1.76)	.74		1.48 (0.69-3.18)			.31		

^a^OR: odds ratio.

^b^Multinomial logistic regression was performed using information exposure of <1 h/day as the reference group. ORs and corresponding 95% CIs are presented as weighted according to the national population and internet users in Thailand. ORs were adjusted for age, sex, marital status, education level, religion, occupation, region of residence, living status, reimbursement scheme, history of mental illness, history of chronic noncommunicable disease, income loss, financial problems, confirmed cases in the community, working from home, quarantine status, fear of COVID-19, and resilient coping.

^c^N/A: not applicable.

### Sensitivity Analysis

Sensitivity analysis was performed by considering the mental health outcomes. A linear relationship was seen between adverse mental health outcomes and the duration of COVID-19-related information exposure, particularly for 3 or more hours per day (see Multimedia Appendix 5, Table S1). Moreover, after using a multivariable ordinal logistic regression model, our findings remained the same compared to those in the primary analysis (see Multimedia Appendix 5, Table S2).

### Data Sharing

Data will be shared upon reasonable request and with permission according to the Health Outcomes and Mental Health Care Evaluation Survey Research Group data release policy.

## Discussion

### Principal Findings

This nationwide online survey was conducted in Thailand. The results indicated that many Thai people used social media as the main source of information during the COVID-19 pandemic. Most participants were exposed to COVID-19-related information for 1 to 2 hours per day. Participants who were exposed to information for 3 or more hours per day had a higher risk of developing depression, anxiety, and insomnia.

Our results were in line with those of a previous study conducted in Wuhan, China, demonstrating that social media exposure was associated with higher odds of developing anxiety and depression [17]. Moreover, previous studies [17,18,20,29] showed that not only the frequency but also the daily duration of exposure to COVID-19-related information was associated with psychosocial problems [29], which corroborated with our study results. The results of a survey conducted in Germany indicated a positive correlation between COVID-19-related media exposure and the severity of nonspecific anxiety and depression [18]. Similar results were reported in a previous study conducted among the Saudi Arabian general population [19].

In essence, during a lockdown or social isolation, people are exposed to a lot of pandemic-related information, especially through social media, which predisposes them to mental health problems. During a severe social disruption, the mass media are expected to satisfy the needs of individuals for information about operationalized guidance to the public, the response of organizations, and an exchange of views with others [30-32]. However, the amount of information that circulated during the COVID-19 pandemic exceeded the information demand. A recent study indicated that repeated media exposure could lead to anxiety due to the effect of vicarious traumatization [31]. Furthermore, media exposure can cause an infodemic, which is defined as fake news, misinformation, and conspiracy theories, making it difficult for individuals to find trusted information [33]. This infodemic may negatively impact an individual’s mental health.

In Thailand, the government issued a national emergency decree, which was put into effect beginning on March 26, 2020, and announced a nationwide curfew on April 3, 2020. People were requested to stay indoors and limit all social contacts. The government also requested that people should wear face masks, practice social distancing, and remain indoors from 10 PM to 4 AM [34]. Due to the implementation of curfews and isolation measures, the number of persons with suspected or confirmed COVID-19 decreased from mid-January to April 6, 2020, which indicated a positive impact of the lockdown on viral transmission reduction and epidemic control [35]. However, the quarantine and isolation measures, coupled with media coverage exposure, increased anxiety and fear in the people, thereby affecting their mental health. Moreover, during the national lockdown and home confinement, the community movement in Thailand had eventually decreased, which resulted in home-based exposure of people to huge amounts of COVID-19-related media information.

Therefore, during a public health crisis, several actions should be taken regarding crisis-related information. First, the media should convey information to the public without sensationalizing the situation and without providing disturbing images to help prevent mental distress. Second, the public should rely on trustworthy sources of information, such as the Centers for Disease Control and Prevention or the WHO, for accurate information. Third, health care providers should play an important role in informing people about practicing protective behaviors [36]. Fourth, public awareness campaigns should be conducted, focusing on the maintenance of mental health in the prevailing situation [37]. Finally, people should spend time with family members who engage in different healthy exercises and sports activities, follow a schedule and routine in daily life, and reduce their time spent on traditional and social media to remain healthy [37]. In terms of mental health policies during the COVID-19 pandemic, the United Nations suggested the application of a whole-of-society approach to promote, protect, and care for an individual’s mental health [38]. Furthermore, public policy solutions would ensure the widespread availability of emergency mental health and psychosocial support. All affected communities require quality mental health services to help their society recover from the effects of the COVID-19 pandemic.

### Strengths and Limitations

This study had several strengths. To our knowledge, this is the first nationwide survey on mental health based on information exposure during the COVID-19 outbreak in Thailand. From a methodological point of view, the analyses were also performed using rigorous statistical approaches to confirm the main findings.

However, our study has some limitations. First, this was a cross-sectional study; therefore, a causal relationship could not be accurately drawn. Second, the survey was conducted online, which is appropriate for short-term evaluation; therefore, the results reflect short-term relationships. Hence, additional longitudinal studies investigating the long-term relationship between mental health and COVID-19-related information exposure are warranted. Third, the survey was conducted over the internet, which is subject to bias due to nonresponse effects and selection bias. Moreover, as information exposure was self-reported, recall bias may be present, and the measures may be influenced by social desirability. Fourth, not everyone in Thailand can access the internet and there could be significant demographic differences between those who can access the internet and those who have limited access to the internet. Thus, selection bias or limited representativeness of the Thai population may be present. Fifth, the majority of the participants were young adults. Only a few older adults participated in the survey; thus, some respondent bias could not be excluded. In addition, the majority of participants (66.4%) were below 30 years of age, which might not reflect the Thai society in its entirety. However, internet usage data in Thailand have demonstrated that workers spend the highest average number of hours surfing the internet per day and account for the highest proportion of internet users [39]. The internet usage data of this group of people may potentially represent the internet consumption of the Thai population. Finally, we lacked information on risk perception and self-care behaviors. This information could help encourage self-improvement and self-management during the COVID-19 pandemic, which may improve an individual’s overall mental health and wellness.

### Conclusions

Our findings from the nationwide online survey indicated a positive association between information exposure during the COVID-19 pandemic and the occurrence of symptoms of depression, anxiety, and insomnia. The strength of the association increased with the duration of media exposure. However, prospective longitudinal studies are needed to investigate the long-term relationship between information exposure and mental health during and after the COVID-19 pandemic.

## Appendix

Multimedia Appendix 1 Patient Health Questionnaire-9 (PHQ-9).

Multimedia Appendix 2 Generalized Anxiety Disorder-7 (GAD-7) scale.

Multimedia Appendix 3 Perceived Stress Scale-10 (PSS-10).

Multimedia Appendix 4 Insomnia Severity Index (ISI).

Multimedia Appendix 5 Sensitivity analysis.

## Abbreviations

GAD-7: Generalized Anxiety Disorder-7
HOME-COVID-19: Health Outcomes and Mental Health Care Evaluation Survey: Under the Pandemic Situation of COVID-19
ISI: Insomnia Severity Index
OR: odds ratio
PHQ-9: Patient Health Questionnaire-9
PSS-10: Perceived Stress Scale-10
WHO: World Health Organization
