# Supplementary material for: Exposure to COVID-19-Related Information and its Association With Mental Health Problems in Thailand: Nationwide, Cross-sectional Survey Study
Source: J Med Internet Res. 2021 Feb 12;23(2):e25363. doi: 10.2196/25363 (PMC7886375; doi:10.2196/25363)
Supplement: Multimedia Appendix 1 [file jmir_v23i2e25363_app1.docx]

**Multimedia Appendix 1:** Patient Health Questionnaire-9 (PHQ-9).

| **Over the last 2 weeks, how often have you been bothered by any of the following problems?** | | **Not at all**  **(0)** | **Several days**  **(1)** | **More than half the days**  **(2)** | **Nearly every day**  **(3)** |
| --- | --- | --- | --- | --- | --- |
| 1. | Little interest or pleasure in doting | 🞏 | 🞏 | 🞏 | 🞏 |
| 2. | Feeling down, depressed, or hopeless | 🞏 | 🞏 | 🞏 | 🞏 |
| 3. | Trouble falling or staying asleep, or sleeping too much | 🞏 | 🞏 | 🞏 | 🞏 |
| 4. | Feeling tried or having little energy | 🞏 | 🞏 | 🞏 | 🞏 |
| 5. | Poor appetite or overeating | 🞏 | 🞏 | 🞏 | 🞏 |
| 6. | Feeling bad about yourself—or that you are a failure or have let yourself or your family down | 🞏 | 🞏 | 🞏 | 🞏 |
| 7. | Trouble concentrating on things, such as reading the newspaper or watching television | 🞏 | 🞏 | 🞏 | 🞏 |
| 8. | Moving or speaking so slowly that other people could have noticed. Or the opposite—being so fidgety or restless that you have been moving around a lot more than usual | 🞏 | 🞏 | 🞏 | 🞏 |
| 9. | Thoughts that you would be better off dead, or of hurting yourself in some way | 🞏 | 🞏 | 🞏 | 🞏 |

Kroenke K, et al. The PHQ-9: Validity of a brief depression severity measure. J Gen Intern Med. 2001;16:606-613.
