# Supplementary material for: Exposure to COVID-19-Related Information and its Association With Mental Health Problems in Thailand: Nationwide, Cross-sectional Survey Study
Source: J Med Internet Res. 2021 Feb 12;23(2):e25363. doi: 10.2196/25363 (PMC7886375; doi:10.2196/25363)
Supplement: Multimedia Appendix 2 [file jmir_v23i2e25363_app2.docx]

**Multimedia Appendix 2:** Generalized Anxiety Disorder-7 (GAD-7) Scale.

| **Over the last 2 weeks, how often have you been bothered by any of the following problems?** | | **Not at all**  **(0)** | **Several days**  **(1)** | **More than half the days**  **(2)** | **Nearly every day**  **(3)** |
| --- | --- | --- | --- | --- | --- |
| 1. | Felling nervous, anxious, or on edge | 🞏 | 🞏 | 🞏 | 🞏 |
| 2. | Not being able to stop or control worrying | 🞏 | 🞏 | 🞏 | 🞏 |
| 3. | Worrying too much about different things | 🞏 | 🞏 | 🞏 | 🞏 |
| 4. | Trouble relaxing | 🞏 | 🞏 | 🞏 | 🞏 |
| 5. | Being so restless that it is hard to sit still | 🞏 | 🞏 | 🞏 | 🞏 |
| 6. | Becoming easily annoyed or irritable | 🞏 | 🞏 | 🞏 | 🞏 |
| 7. | Feeling afraid, as if something awful might happen | 🞏 | 🞏 | 🞏 | 🞏 |

Spitzer RL, et al. A brief measure for assessing generalized anxiety disorder: the GAD-7. Arch Intern Med. 2006;166:1092.
