# Supplementary material for: Exposure to COVID-19-Related Information and its Association With Mental Health Problems in Thailand: Nationwide, Cross-sectional Survey Study
Source: J Med Internet Res. 2021 Feb 12;23(2):e25363. doi: 10.2196/25363 (PMC7886375; doi:10.2196/25363)
Supplement: Multimedia Appendix 3 [file jmir_v23i2e25363_app3.docx]

**Multimedia Appendix 3:** Perceived Stress Scale-10 (PSS-10).

| **In the last month** | | **Never**  **(0)** | **Almost never**  **(1)** | **Some-times (2)** | **Fairly often (3)** | **Very often**  **(4)** |
| --- | --- | --- | --- | --- | --- | --- |
| 1. | How often have you been upset because of something that happened unexpectedly? | 🞏 | 🞏 | 🞏 | 🞏 | 🞏 |
| 2. | How often have you felt that you were unable to control the important things in your life? | 🞏 | 🞏 | 🞏 | 🞏 | 🞏 |
| 3. | How often have you felt nervous and stressed? | 🞏 | 🞏 | 🞏 | 🞏 | 🞏 |
| 4. | How often have you felt confident about your ability to handle your personal problems? | 🞏 | 🞏 | 🞏 | 🞏 | 🞏 |
| 5. | How often have you felt that things were going your way? | 🞏 | 🞏 | 🞏 | 🞏 | 🞏 |
| 6. | How often have you found that you could not cope with all the things that you had to do? | 🞏 | 🞏 | 🞏 | 🞏 | 🞏 |
| 7. | How often have you been able to control irritations in your life? | 🞏 | 🞏 | 🞏 | 🞏 | 🞏 |
| 8. | How often have you felt that you were on top of things? | 🞏 | 🞏 | 🞏 | 🞏 | 🞏 |
| 9. | How often have you been angered because of things that happened that were outside of your control? | 🞏 | 🞏 | 🞏 | 🞏 | 🞏 |
| 10. | How often have you felt difficulties were piling up so high that you could not overcome them? | 🞏 | 🞏 | 🞏 | 🞏 | 🞏 |

Cohen S, Williamson G: Perceived stress in a probability sample of the United States. The Social Psychology of Health: Claremont Symposium on Applied Social Psychology. Edited by: Spacapan S, Oskamp S. 1988, Newbury Park, CA: Sage, 31-67.
