# Supplementary material for: Exposure to COVID-19-Related Information and its Association With Mental Health Problems in Thailand: Nationwide, Cross-sectional Survey Study
Source: J Med Internet Res. 2021 Feb 12;23(2):e25363. doi: 10.2196/25363 (PMC7886375; doi:10.2196/25363)
Supplement: Multimedia Appendix 4 [file jmir_v23i2e25363_app4.docx]

**Multimedia Appendix 4:** Insomnia Severity Index (ISI).

| **Please rate the current (i.e. last two weeks) severity of your insomnia problem(s)** | | | | | | |
| --- | --- | --- | --- | --- | --- | --- |
|  | **Insomnia Problem** | **None**  **0** | **Mild**  **1** | **Moderate**  **2** | **Severe**  **3** | **Very severe**  **4** |
| 1. | Difficult falling asleep | 🞏 | 🞏 | 🞏 | 🞏 | 🞏 |
| 2. | Difficulty staying asleep | 🞏 | 🞏 | 🞏 | 🞏 | 🞏 |
| 3. | Problems waking up too early | 🞏 | 🞏 | 🞏 | 🞏 | 🞏 |
| 4. | How satisfied/dissatisfied are you with your current sleep pattern? | | | | | |
|  | 🞏 Very satisfied (0)  🞏 Satisfied (1) | | 🞏 Moderately satisfied (2)  🞏 Dissatisfied (3)  🞏 Very dissatisfied (4) | | | |
| 5. | How noticeable to others do you think your sleep problem is in terms of impairing the quality of your life? | | | | | |
|  | 🞏 Not at all noticeable (0)  🞏 A little (1) | | 🞏 Somewhat (2)  🞏 Much (3)  🞏 Very much noticeable (4) | | | |
| 6. | How worried/distressed are you about your current sleep problem? | | | | | |
|  | 🞏 Not at all worried (0)  🞏 A little (1) | | 🞏 Somewhat (2)  🞏 Much (3)  🞏 Very much worried (4) | | | |
| 7. | To what extent do you consider your sleep problem to interfere with your daily functioning (e.g. daytime fatigue, mood, ability to function at work/daily chores, concentration, memory, mood, etc.) currently? | | | | | |
|  | 🞏 Not at all interfering (0)  🞏 A little (1) | | 🞏 Somewhat (2)  🞏 Much (3)  🞏 Very much interfering (4) | | | |

Morin CM, et al. The Insomnia Severity Index: psychometric indicators to detect insomnia cases and evaluate treatment response. Sleep 2011;34(5):601-608.
