# Supplementary material for: Exposure to COVID-19-Related Information and its Association With Mental Health Problems in Thailand: Nationwide, Cross-sectional Survey Study
Source: J Med Internet Res. 2021 Feb 12;23(2):e25363. doi: 10.2196/25363 (PMC7886375; doi:10.2196/25363)
Supplement: Multimedia Appendix 5 [file jmir_v23i2e25363_app5.docx]

**Multimedia Appendix 5.** Sensitivity analysis.

**Table S1.** Mental Health Outcomes According to COVID-19-Related Information Exposure Duration: Multivariable Linear Regression Model^†^

| **COVID-19-Related Information Exposure Duration** | **Depression: Lower=Better** | | | **Anxiety: Lower=Better** | | | **Stress: Lower=Better** | | | **Insomnia: Lower=Better** | | |
| --- | --- | --- | --- | --- | --- | --- | --- | --- | --- | --- | --- | --- |
|  | **Mean (min-max)** | **Adjusted Difference (95% CI)** | ***P* Value** | **Mean (min-max)** | **Adjusted Difference (95% CI)** | ***P* Value** | **Mean (min-max)** | **Adjusted Difference (95% CI)** | ***P* Value** | **Mean (min-max)** | **Adjusted Difference (95% CI)** | ***P* Value** |
| <1 h/day | 7.6  (0-27) | Reference (1.00) |  | 4.2 (0-21) | Reference (1.00) |  | 16.9  (0-39) | Reference (1.00) |  | 8.2  (0-28) | Reference (1.00) |  |
| 1-2 h/day | 8.1  (0-27) | 0.39  (-0.14, 0.91) | 0.147 | 4.7 (0-21) | 0.77  (0.36, 1.18) | <0.001 | 17.4  (0-40) | 0.55  (0.01, 1.09) | 0.045 | 8.8  (0-28) | 0.25  (-0.26, 0.77) | 0.337 |
| ≥3 h/day | 8.8  (0-27) | 1.22  (0.59, 1.86) | <0.001 | 5.6 (0-21) | 1.70  (1.15, 2.25) | <0.001 | 18.2  (0-40) | 1.39  (0.74, 2.05) | <0.001 | 9.3  (0-25) | 1.06  (0.43, 1.69) | 0.001 |

Noted: the adjusted difference corresponding to 95% CIs are presented weighted according to the national population and internet users in Thailand.

^†^Adjusted for age, sex, marital status, education level, religion, occupation, region of residence, living status, reimbursement scheme, mental illness history, chronic non-communicable disease history, income loss, financial problems, confirmed cases in the community, working from home, quarantine status, fear of COVID-19, and resilient coping.

Abbreviations: CI, confidence interval; COVID-19, coronavirus disease-2019; GAD-7, Generalized Anxiety Disorder-7; ISI, Insomnia Severity Index; OR, odds ratio; PHQ-9, Patient Health Questionnaire-9; PSS-10, Perceived Stress Scale-10.

**Table S2.** Multivariable Ordinal Logistic Regression Model Results of COVID-19-Related Information Exposure Duration and the Severity of Mental Health Problems^†^

| **COVID-19-Related Information Exposure Duration** | **Depression: PHQ-9** | | **Anxiety: GAD-7** | | | **Stress: PSS-10** | | | **Insomnia: ISI** | |
| --- | --- | --- | --- | --- | --- | --- | --- | --- | --- | --- |
|  | **Estimate Adjusted OR (95% CI)** | ***P* Value** | **Estimate Adjusted OR** **(95% CI)** | ***P* Value** | **Estimate Adjusted OR** **(95% CI)** | | ***P* Value** | **Estimate Adjusted OR** **(95% CI)** | | ***P* Value** |
| <1 h/day | Reference (1.00) |  | Reference (1.00) |  | Reference (1.00) | |  | Reference (1.00) | |  |
| 1-2 h/day | 1.13  (0.91 – 1.39) | 0.259 | 1.34  (1.09 – 1.66) | 0.005 | 1.09  (0.88 – 1.35) | | 0.423 | 1.10  (0.90 – 1.34) | | 0.362 |
| ≥3 h/day | 1.49  (1.15 – 1.92) | 0.002 | 2.19  (1.70 – 2.84) | <0.001 | 1.26  (0.97 – 1.62) | | 0.076 | 1.49  (1.18 – 1.88) | | 0.001 |

Noted: ordinal logistic regression using no/minimal mental health as the reference group; and the adjusted difference corresponding to 95% CIs are presented weighted according to the national population and internet users in Thailand.

^†^Adjusted for age, sex, marital status, education level, religion, occupation, region of residence, living status, reimbursement scheme, mental illness history, chronic non-communicable diseases history, income loss, financial problems, confirmed cases in the community, working from home, quarantine status, fear of COVID-19, and resilient coping.

Abbreviations: CI, confidence interval; COVID-19, coronavirus disease-2019; GAD-7, Generalized Anxiety Disorder-7; ISI, Insomnia Severity Index; OR, odds ratio; PHQ-9, Patient Health Questionnaire-9; PSS-10, Perceived Stress Scale-10.
